# An Enhanced Indoor Positioning Technique Based on a Novel Received Signal Strength Indicator Distance Prediction and Correction Model

**DOI:** 10.3390/s21030719

**Published:** 2021-01-21

**Authors:** Mohammed Nagah Amr, Hussein M. ELAttar, Mohamed H. Abd El Azeem, Hesham El Badawy

**Affiliations:** 1 Department of Electronics and Communications Engineering, Canadian International College (CIC), Cairo 12588, Egypt; moh_nagah@cic-cairo.com; 2 Department of Electronics and Communications Engineering, Arab Academy for Science, Technology and Maritime Transport (AASTMT), Cairo 11799, Egypt; hattar@aast.edu (H.M.E.); mhabdazeem@aast.edu (M.H.A.E.A.); 3 Network Planning Department, National Telecommunication Institute (NTI), Cairo 11432, Egypt

**Keywords:** indoor positioning, RSSI, correction factor, Bluetooth Low Energy, beacon, enhanced centroid positioning algorithm

## Abstract

Indoor positioning has become a very promising research topic due to the growing demand for accurate node location information for indoor environments. Nonetheless, current positioning algorithms typically present the issue of inaccurate positioning due to communication noise and interferences. In addition, most of the indoor positioning techniques require additional hardware equipment and complex algorithms to achieve high positioning accuracy. This leads to higher energy consumption and communication cost. Therefore, this paper proposes an enhanced indoor positioning technique based on a novel received signal strength indication (RSSI) distance prediction and correction model to improve the positioning accuracy of target nodes in indoor environments, with contributions including a new distance correction formula based on RSSI log-distance model, a correction factor (Beta) with a correction exponent (Sigma) for each distance between unknown node and beacon (anchor nodes) which are driven from the correction formula, and by utilizing the previous factors in the unknown node, enhanced centroid positioning algorithm is applied to calculate the final node positioning coordinates. Moreover, in this study, we used Bluetooth Low Energy (BLE) beacons to meet the principle of low energy consumption. The experimental results of the proposed enhanced centroid positioning algorithm have a significantly lower average localization error (ALE) than the currently existing algorithms. Also, the proposed technique achieves higher positioning stability than conventional methods. The proposed technique was experimentally tested for different received RSSI samples’ number to verify its feasibility in real-time. The proposed technique’s positioning accuracy is promoted by 80.97% and 67.51% at the office room and the corridor, respectively, compared with the conventional RSSI trilateration positioning technique. The proposed technique also improves localization stability by 1.64 and 2.3-fold at the office room and the corridor, respectively, compared to the traditional RSSI localization method. Finally, the proposed correction model is totally possible in real-time when the RSSI sample number is 50 or more.

## 1. Introduction

One of the essential elements of contextual information is the position of a user or device in a given space. The widespread use of sensors, smartphones and the mobile internet has allowed precise positioning in real-time, and this approach is being extended slowly to different situations [1]. A secure, user-friendly and precise navigation location information method for mobile applications could open the door to many innovative applications and create opportunities for new businesses. Therefore, it is considered a milestone in the realization of the Internet of Things (IoT). For example, location-based services, ambient assisted living (AAL), health applications, robotics, and cellular network- based indoor positioning are promising applications to achieve complete navigation systems [2].

This demand for indoor localization for universal mobile computing has led to impressive amount of research in the past few years. Some of the fundamental research involved specific sensors and emitters installed inside buildings to position people and objects (infrastructure-based indoor positioning). Though very accurate, this method is not scalable for business deployments and includes a specific overhead cost to install and maintain the extra infrastructure. A different popular method employs the existing infrastructure utilizing wireless access points to locate and triangulate using mobile devices. This approach is very accurate, but it normally needs a comprehensive surveying and training effort to establish a radio frequency (RF) map of the building. There are further enhancements to this method, which decrease the efforts or discharge them entirely but at the expense of accuracy. With mobile technology growing more powerful over the last few years, it is presently embedded with more sensors, promoting the prediction’s accuracy by merging them with certain earlier technologies without any support from the existing infrastructures (infrastructure-less indoor positioning).

Different technologies such as ultra-wideband [3], Bluetooth [4], wireless local area networks (WLANs) [5], radio frequency identification (RFID) [6], micro-electro-mechanical (MEMS) [7], magnetic field [8], ultrasonic [9], computer vision [10], infrared signal [11] and other techniques have been used for indoor localization. However, Bluetooth low-power sources are preferred in indoor positioning due to their advantages, including fast installation, low power consumption and low cost. Apple introduced the Bluetooth low-energy (BLE)-based iBeacon technology and this paved the way for BLE to be widely used in different indoor environments [12]. In this work, the proposed positioning method depends on BLE-based beacons as a stable signal source with low cost and energy consumption sources.

Measuring the distance between the unknown node and beacons is an essential part of the positioning process within indoor environments. Most of the existing node localization algorithms used nowadays can be divided into two categories depending on whether distance measurements are required or not. One of these categories is the range-free measurement localization algorithm and the other is the range-based measurement localization algorithm [13,14]. The distance measurement algorithm calculates the distance between the known beacon node and the unknown node connected to it, utilizing their communication link parameters. The main categories of distance measurement algorithms are the angle of arrival (AOA) based-algorithm [15,16], time of arrival (TOA) based-algorithm, time difference of arrival (TDOA) based-algorithm [17,18] and the received signal strength indication (RSSI) based-algorithm [19,20,21,22,23]. In the previously mentioned algorithms, the TOA, TDOA, and AOA need to correctly determine the distance between the unknown target node and the specified beacon node by using a high-complexity algorithm that requires high energy consumption and additional hardware. Each of these considerably increases the communication cost of the positioning system. Therefore, the proposed indoor positioning technique is based on RSSI distance measurement algorithm aiming at a cost-effective indoor positioning solution without neglecting the positioning accuracy.

One of the essential procedures to accomplish the positioning process is the positioning algorithm. As previously mentioned, the measurement distance algorithm estimates the actual distance between the unknown node and the beacon. The positioning algorithm is responsible for calculating the final coordinates of the unknown node by using the distance measurements between the beacon and the unknown node. The positioning algorithm can be constructed by using several methods. A centroid localization algorithm based on the distance or angle information between the unknown node and the beacon is presented in [24,25,26,27]. However, this algorithm produces a large positioning error as the node distribution issue is not fully considered in addition to the presence of severe fluctuation in distance measurements. Another algorithm based on the centroid algorithm called the weighted centroid algorithm is developed in [28,29,30,31]. In this algorithm, a weighting factor is introduced to compensate for the error in estimated distance from measurements. Nevertheless, the positioning error is still relatively high, especially in modern indoor architectures. In [32], a fingerprint-based positioning algorithm is proposed by collecting RSSI samples in the fingerprint database. In [33], the authors have executed and analyzed several positioning algorithms (centroid localization, proximity localization, weighted centroid localization, weight-compensated weighted centroid localization based on RSSI, fingerprinting, and trilateration localization). The authors also suggested and performed a fuzzy logic-based system to choose the most suitable algorithm depending upon the room’s area, the beacon’s number available, and the signal strength. They concluded that the fingerprinting positioning algorithm is the most fitting one. In [34], the authors implemented a fingerprinting algorithm with fuzzy logic type-2 suitable for employment as an indoor positioning method with BLE beacons with ALE of 0.43 m, but as fingerprint positioning technology methods need several additional algorithms to support them, thus the computational resource consumption and algorithmic program complexity are comparatively high. Also, this methodology needs a large amount of a priori information support which adds a high-cost issue. Besides, the RF signal may suffer from multipath effects and electromagnetic interference in complex indoor environments.

To address the low positioning accuracy problem, many researchers have introduced the hybrid positioning solutions. A hybrid positioning technique using hybrid metrics including time-of-flight (ToF) and angle-of-arrival (AoA) which are combined with the RSS fingerprinting system is presented in [35]. Another hybrid positioning method based on Bluetooth beacons, geomagnetic field, inertial measurement unit (IMU) sensors, and smartphone cameras is presented in [36]. These methods enhance the localization accuracy, but hybrid positioning techniques require additional hardware equipment which increases the communication cost. For low cost and high positioning accuracy, an iterative centroid positioning algorithm based on an RSSI distance model is presented in [37]. In this algorithm, a distance deviation coefficient was driven to correct the distance iteratively based on the noise impact factor which is considered a better theoretical approach to improving positioning accuracy. Nonetheless, the positioning results based on the assumption that the noise impact factor is the same among all beacons is an impractical assumption as the RSSI has time-varying characteristics.

Aiming at addressing the previously mentioned issues, this paper presents an improved indoor positioning technique based on a new RSSI distance prediction and correction model. The proposed technique’ correction factors are based on a novel method for collecting and obtaining the correction factors, which improves both the localization accuracy and stability. Moreover, the proposed technique does not require any additional hardware; hence it can be considered a cost-effective, low energy consumption solution for indoor localization. Our proposed method is experimentally tested and verified in an indoor office environment and a corridor using BLE beacons. In this paper, our contributions may be summarized in the following parts:

- A new distance prediction and correction formula is introduced. The correction factors are based on RSSI log-distance distribution model. A large number of RSSI samples are collected and each RSSI sample is converted into its equivalent distance based on the logarithmic relationship between received signal strength and distance. The correction factors are driven from the RSSI-distances samples rather than the RSSI samples themselves. We proved that this approach has more measurement stability, which leads to higher positioning stability results with improved accuracy.
- A correction factor and a correction exponent for different distances are driven from the correction formula at each beacon. First, the correction factor for a certain distance is determined by calculating the mean and the median of RSSI samples equivalent distances. Then, the correction exponent is driven for each distance from the correction formula. Each beacon has its correction factors for certain distances according to the indoor environment area.
- At the unknown terminal, the calculated correction factors for each beacon are stored in a lookup table. By utilizing those factors on the unknown node, an enhanced centroid positioning algorithm is applied by correcting the estimated distance from the real-time RSSI samples and finally calculate the real coordinates of the unknown node. The algorithm does not consume much time to find the position of the unknown terminal. Thus, complexity is relatively lower. The experimental results of our method show improvement in both positioning accuracy and stability compared to other existed algorithms.

The remainder of this paper is organized as follows: Section 2 describes our positioning technique. In Section 3, we illustrate the implementation of our experiment and the software and hardware devices used in experiments. In Section 4, we present our positioning results compared to other algorithms and conventional methods. Finally, Section 5 presents the conclusions of the paper and suggestions for future work.

## 2. Methods

This section presents the RSSI log-distance model and the positioning methods used in this work. Section 4 discusses the positioning results of the proposed technique.

### 2.1. RSSI Distance Model

The main concept of RSSI ranging method is to measure the distance between the receiving signal node and the transmitting signal node by measuring the received signal strength as the propagation loss affects the transmitted wireless signal. The Bluetooth signal propagation model follows the log-distance distribution model [38,39,40], which described by:

(1) $$P_{L}\left(d\right)=P_{L}\left(d_{0}\right)+10n\;log\left(\frac{d}{d_{0}}\right)+X_{\delta }$$

where $P_{L}\left(d\right)$ is the RSSI at the receiving node separated from the transmitting source by distance d; $P_{L}\left(d_{0}\right)$ is the RSSI at the receiving node separated from the source by a reference distance $d_{0};$ n is the path-loss propagation exponent which takes different values depending on the surrounding wireless transmission environment; $X_{\delta }$ is a Gaussian random variable with zero mean and $\sigma^{2}$ variance. It worth mentioning that the measurement error in RSSI does not regularly produce a Gaussian distribution. However, by using approximation (curve fitting), the RSSI measurement error is treated as a gaussian random variable for simplicity. consequently, the reference distance is usually taken as one meter, and the equation is simplified as follows:

(2) RSSI=A−10nlog(d)

where RSSI is the signal strength at a distance d from the transmitting source, and A refers to signal strength at 1 m distance from the signal source. The distance can be expressed from Equation (2) as follows:

(3) $$d=10^{\frac{A-RSSI}{10n}}$$

### 2.2. RSSI Distance Prediction and Correction Model

The emitted RSS from the BLE beacons is affected by several factors such as multipath effects (reflection, refraction) and absorption from water bodies. Moreover, channel hopping influencing the RSS values since the advertisement packages are sent in three different channels. Therefore, it is challenging to calculate the actual distance using the RSSI log-distance distribution model because severe RSSI fluctuation may occur especially in complex indoor environments. To minimize the localization error introduced by RSSI fluctuation, a new RSSI distance prediction and correction model is proposed in this paper. To predict the real distance, 5000 RSSI samples are collected (95% confidence interval) at 1 m, 2 m, 3 m, 4 m, 5 m and 6 m distances from each Bluetooth beacon. A series of signal strength values can be given as follows:

(4) $$RSSI=\left\{rssi_{i}\right\}\;\left(i=1,2,3,\ldots ,5000\right),$$

Each RSSI sample is converted to its equivalent distance using RSSI log-distance relationship in Equation (3). A series of distance values can be given as follows:

(5) $$D=\left\{d_{i}\right\}\;\left(i=1,2,3,\ldots ,5000\right),$$

RSSI fluctuation is an indispensable concern for wireless signals. Therefore reducing the variance range for measurements is vital for improving the stability and accuracy of the positioning. To achieve this, we calculated the standard deviation of the 5000 collected RSSI samples and their 5000 equivalent distances, as shown in Table 1.

**Figure 1:**
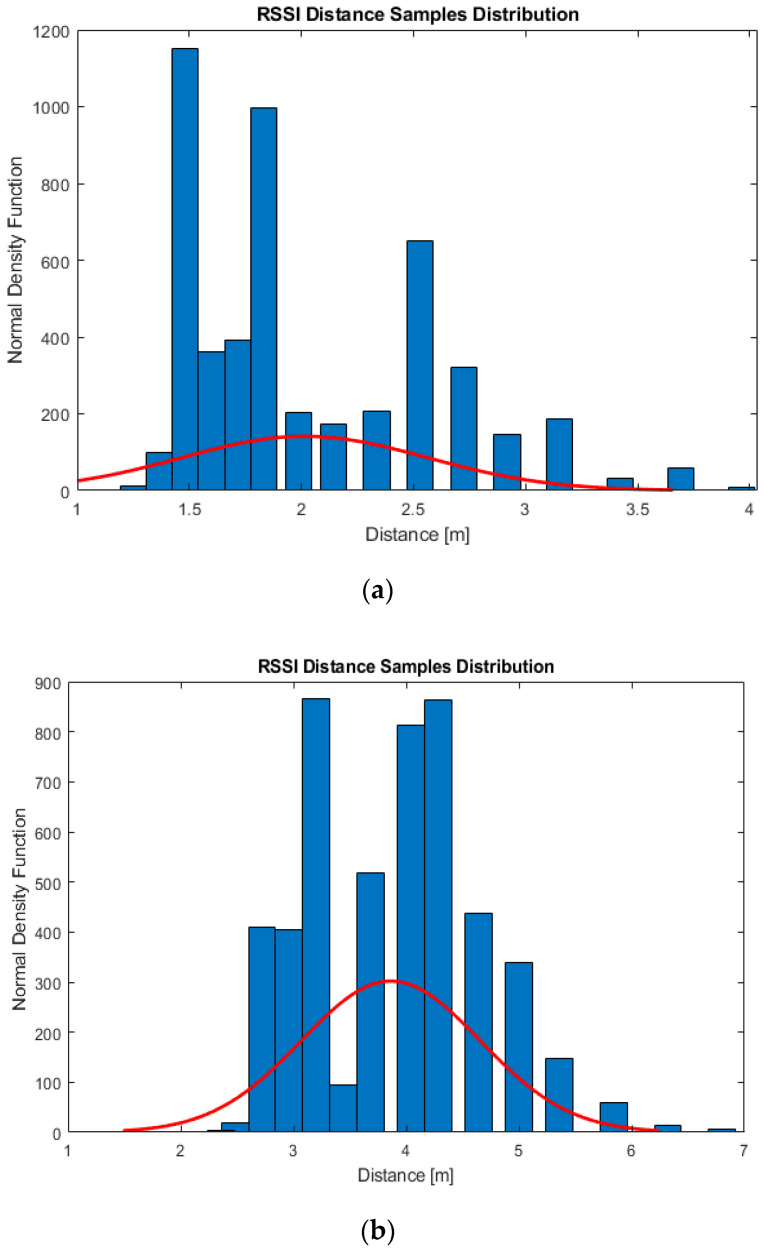
The distribution of RSSI distance samples fitted to the normal density function at: (**a**) 1 m where (*D_mean_* > *D_median_*); (**b**) 2 m where $\left(D_{mean}<D_{median}\right)$.

As presented in Table 1, the standard deviation of distance samples is smaller than that of RSSI samples. Hence, the correction model is based on the equivalent distances rather than RSSI samples itself as it is considered a more stable measurement value. It is necessary to define a characteristic quantity to represent the overall node distance measurement. The two statistical parameters, the mean value and the median of distance measurements, can be closely used to denote the overall distance measurement. The mean and the median values of the distances are calculated as shown in Equations (6) and (7):

(6) $$D_{mean}=\frac{1}{n}\overset{n}{\underset{i=1}{\sum }}d_{i}\left(n=5000\right),$$

(7) $$D_{median}=\frac{1}{2}\left(D_{s_{⎣\left(n+1\right)/2⎦}}+D_{s_{⎡\left(n+1\right)/2⎤}}\right)\left(n=5000\right),$$

where $D_{s}$ is an ordered list of the i distance values in Equation (5), and ⎣.⎦ and ⎡.⎤ are the floor and ceiling functions, respectively. After calculating the previous values, the training stage of data is finished. Now we introduce the prediction formula as follows:

(8) $$D_{predicted}=\{\begin{array}{c} D_{mean}\cdot \beta^{\sigma },|D_{mean}\leq D_{median}\\ \;D_{median}\cdot \beta^{\sigma },|D_{mean}>D_{median} \end{array}$$

where $D_{predicted}$ is the predicted distance based on 5000 RSSI samples; β is the correction factor which should have a specific value for 1, 2, 3, 4, 5 and 6 m distances, and its value is defined in Equation (9); σ is the correction exponent of the correction factor which also should have a specific value for the chosen distances. The 5000 RSSI samples are collected for each beacon independently at 1, 2, 3, 4, 5 and 6 m distances, which means 30,000 RSSI samples are collected from each beacon. The experiments are carried out on two experimental sites, and each site has four beacons, which result in a total of 240,000 collected RSSI samples. Based on extensive experimental measurements of 240,000 RSSI samples, the values of $D_{mean}$ and $\;D_{median}$ are found to be always larger than the real distance value. However, $D_{mean}$ and $D_{median}$ inequality is not certain for all the distances. Figure 1 shows the distribution of RSSI distance samples fitted to the normal density function at 1 m $\left(D_{mean}>D_{median}\right)$ and 2 m $\left(D_{mean}<D_{median}\right)$ distances.

Therefore, the smaller value between $D_{mean}$ and $D_{median}$ is taken as a primary distance to be corrected as mentioned in Equation (8):

(9) $$\beta =\{\begin{array}{c} \;\frac{D_{mean}}{D_{median}},D_{mean}\leq D_{median}\\ \frac{D_{median}}{D_{mean}},D_{mean}>D_{median} \end{array}$$

As indicated in Equation (9), the value of the correction factor β is chosen based on the values of $D_{mean}$ and $D_{median}$ to be less than unity to fit the fact that the measured RSSI distance is always larger than the real distance. The distance correction factor β is considered as a deviation factor between $D_{mean}$ and $D_{median}$, which can be used to correct one of them iteratively as in [37]. However, the iterations criteria require additional RSSI real-time processing and more complexity. Therefore, the correction exponent σ is introduced in Equation (8) to compensate for the number of iterations required to correct $D_{mean}$ and $D_{median}$. Accordingly, the correction computations are done once for each distance. In order to derive the appropriate value of the correction exponent, $D_{predicted}$ is replaced with the real distance values which are 1, 2, 3, 4, 5 and 6 m, respectively, so Equation (8) can be written as:

(10) $$D_{real}=\{\begin{array}{c} D_{mean}\cdot \beta^{\sigma },|D_{mean}\leq D_{median}\\ \;D_{median}\cdot \beta^{\sigma },|D_{mean}>D_{median} \end{array}$$

(11) $$\beta^{\sigma }=\{\begin{array}{c} \frac{D_{real}}{D_{mean}},|D_{mean}\leq D_{median}\\ \frac{D_{real}}{D_{median}},|D_{mean}>D_{median} \end{array}$$

(12) $$\sigma \log\beta =\{\begin{array}{c} \log\left(\frac{D_{real}}{D_{mean}}\right),|D_{mean}\leq D_{median}\\ \log\left(\frac{D_{real}}{D_{median}}\right),|D_{mean}>D_{median} \end{array}$$

(13) $$\sigma =\{\begin{array}{c} \log\left(\frac{D_{real}}{D_{mean}}\right)/\log\beta ,|D_{mean}\leq D_{median}\\ \log\left(\frac{D_{real}}{D_{median}}\right)\;/\log\beta ,|D_{mean}>D_{median} \end{array}$$

**Figure 2:**
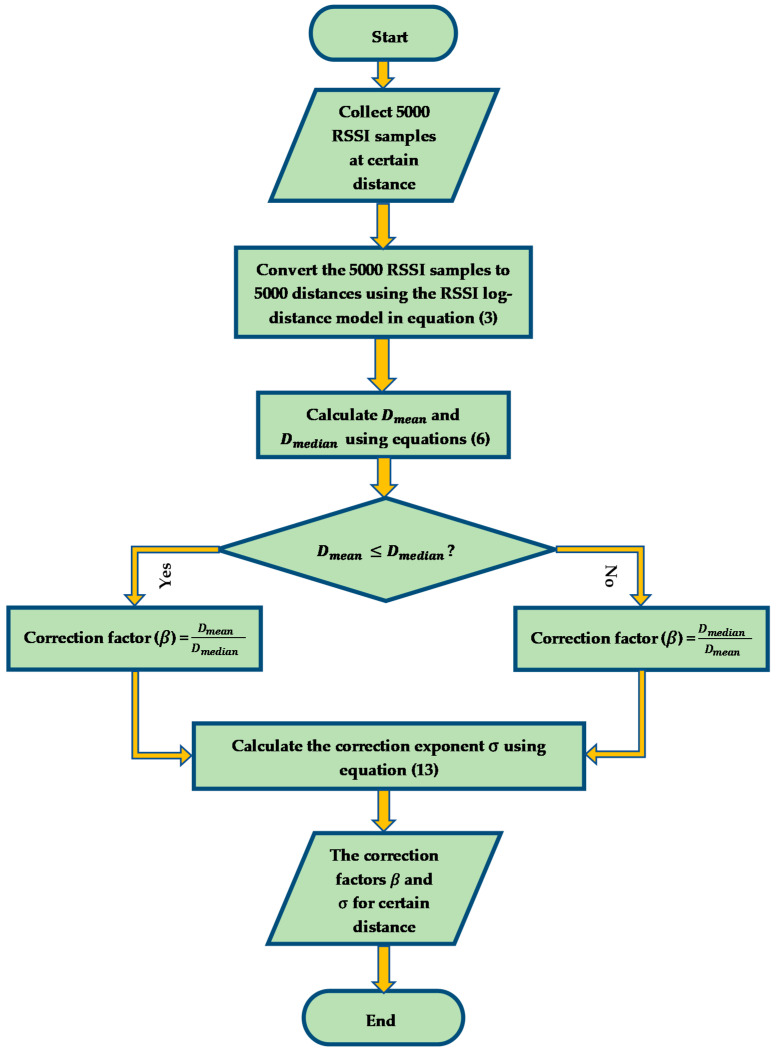
A flow chart of the RSSI distance prediction and correction algorithm for a certain distance.

Equation (13) represents an expression of the correction exponent for different distances. In this work, we found the correction factor and correction exponent at 1, 2, 3, 4, 5 and 6 m distances for each beacon to fit the experimental areas’ size. The proposed relationship for the correction factor and correction exponent may be considered as an asymptotic correction formula based on our experiments and measurements that extended to 240,000 RSSI samples. We found that this approximation is some sort of reasonable fitness to the correction of the indoor localization problem. We justify these values based on the proposed relation, and we predict its accuracy in the proposed experimental areas. In addition, the proposed equations are trying to correlate between the expectation of the measured values versus the accumulation of the readings so that we can predict (approximately) the real distance. In other words, by using the proposed relationship, we may approach a good estimation of the concurrent real distance between the unknown node and the beacon in the indoor application and we can claim that this approximation is suited enough for indoor application and localization scenario. Figure 2 summarizes the process of our RSSI distance prediction and correction algorithm.

It is very important to mention that the process of collection of RSSI samples at certain distances per beacon was performed in the same experimental environment where we test our enhanced positioning centroid algorithm. In other words, the effects of electromagnetic interference in the indoor environment are considered when the correction factor and correction exponent are derived. Hence, the positioning process is performed more accurately.

### 2.3. The Triangle Centroid Positioning Algorithm

**Figure 3:**
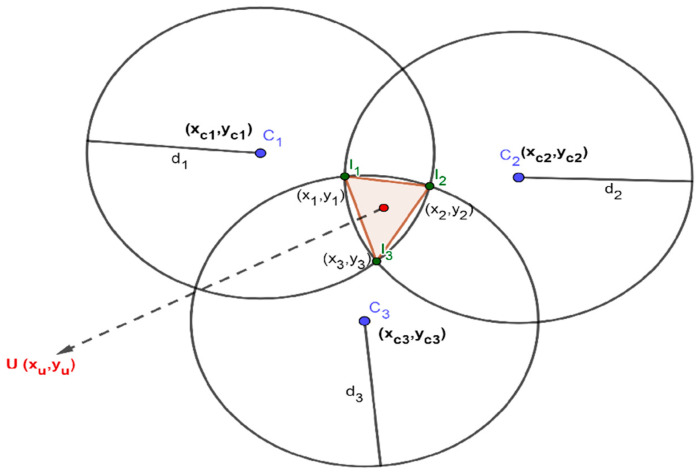
A schematic diagram of the triangle centroid localization algorithm [24,25,26,27].

The main concept of the triangle centroid localization algorithm is described as follows: three beacons are placed where their coordinates represent the centers of three circles and the distance between each beacon and the unknown node is treated as a radius of each circle. The three circles are intersecting in six points where a triangle is formed from the inner intersection points. Then, the unknown node coordinates can be found by calculating the centroid of the triangle. Figure 3 shows a schematic diagram of the triangle centroid localization algorithm.

As depicted in Figure 3, C_1_, C_2,_ and C_3_ are defined as the three beacons positions with coordinates of $\left(x_{c1},y_{c1}\right),\;\left(x_{c2},y_{c2}\right)$ and $\left(x_{c3},y_{c3}\right)$ as centers of circles and their corresponding radiuses are d_1_, d_2,_ and d_3_, respectively. The points $I_{1}\left(x_{1},y_{1}\right),\;I_{2}\left(x_{2},y_{2}\right)$ and $I_{3}\left(x_{3},y_{3}\right)$ are the three inner intersection points of the three circles which are considered as the vertexes of the triangle centroid localization algorithm.

The coordinates of the intersection point between the circles C_2_ and C_3_ can be obtained by solving Equation (14):

(14) $$\{\begin{array}{c} \;{\left(x-x_{c2}\right)}^{2}+{\left(y-y_{c2}\right)}^{2}=\;d_{2}^{2}\;\\ {\left(x-x_{c3}\right)}^{2}+{\left(y-y_{c3}\right)}^{2}=\;d_{3}^{2} \end{array}\;$$

By solving the two sets of coordinates in Equation (14), the coordinates of the inner intersection point $I_{1}\left(x_{1},y_{1}\right)$ can be obtained. The remaining inner intersection points $I_{2}\left(x_{2},y_{2}\right)$ and $I_{3}\left(x_{3},y_{3}\right)$ is calculated similarly as the point $I_{1}\left(x_{1},y_{1}\right)$.

Finally, the coordinates of the unknown node $U\left(x_{u},y_{u}\right)$ can be calculated using Equation (15):

(15) {xu=1k∑i=1kxi yu=1k∑i=1kyi (k=3),

### 2.4. The Weighted Centroid Positioning Algorithm

As mentioned earlier in this paper, the centroid localization algorithm does not fully consider the node distribution issue, which leads to high positioning errors. By taking the geometric distribution of the beacon nodes into consideration, the weighted centroid localization algorithm is developed in [28,29,30,31]. A new weighting factor is introduced to reflect the impact of each beacon node on the centroid position. In this paper, we used the weighting factors as in [25] where the weighting factor of the inner intersection point equals the reciprocal of the radiuses of the intersecting circles at that point. Referring to Figure 3, the weighting factors can be expressed as follows:

(16) $$\{\begin{array}{c} w_{1}=\frac{1}{d_{2}+d_{3}}\\ w_{2}=\frac{1}{d_{1}+d_{3}}\\ w_{3}=\frac{1}{d_{1}+d_{2}} \end{array}\;,$$

where $w_{1},\;w_{2}$ and $w_{3}$ are the weighting factors of the three inner intersection points $I_{1}\left(x_{1},y_{1}\right),\;I_{2}\left(x_{2},y_{2}\right)$ and $I_{3}\left(x_{3},y_{3}\right)$, respectively. The final coordinates of the unknown node $U\left(x_{u},y_{u}\right)$ can be obtained using Equation (17):

(17) {xu=1k∑i=1kwixiwi yu=1k∑i=1kwiyiwi (k=3),

### 2.5. The Iterative Centroid Positioning Algorithm

The iterative centroid localization algorithm is presented in [37] and it is based on RSSI log-distance distribution model. As we use the iterative centroid algorithm in the comparison between our enhanced centroid algorithm and the other localization algorithms, we will briefly summarize the main principle of it in the following steps:

1. After receiving RSSI samples from the beacons, The blind (unknown node) chooses the highest three RSSI values and calculate their distances from log-distance model using Equation (3), recall them $d_{p1,2,3}$; where $d_{p1}$ is the calculated distance between the unknown node and one of the closest beacons to it using the log-distance distribution model.
2. The distances between the three beacons and the unknown node are used in the triangle centroid positioning algorithm to calculate the initial coordinates of the unknown node. Then the calculated coordinates of the unknown node are used to find the distance between it and the three beacon nodes, recall them $d_{c1,2,3};$ where $d_{c1}$ is the calculated distance between the unknown node and one of the closest beacons to it using the triangle centroid positioning algorithm.
3. To measure the deviation between the RSSI log-distance model calculated distances and the triangle centroid localization algorithm calculated distances, a distance deviation coefficient is introduced using Equation (18);
  (18) $$C_{dev}=\frac{d_{p1,2,3}}{d_{c1,2,3}}\;,$$
4. The distance deviation coefficients are sorted to find the median value of them, recall it $C_{m-dev};$ where $C_{m-dev}$ is the median distance deviation coefficient, which is used to correct the log-distance model calculated distances.
5. The corrected distances can be obtained by using Equation (19):
  (19) $$d_{n1,2,3}=\frac{d_{p1,2,3}}{c_{m-dev}}\;,$$
6. The corrected distances are inserted into the triangle centroid localization algorithm iteratively starting from step 2 until a termination condition is satisfied. Since the iteration error no longer varies after 10 iterations, we set the number of iterations to be 10 in our comparative analysis.

### 2.6. The Proposed Enhanced Centroid Positioning Algorithm

In this subsection, we present our enhanced centroid positioning algorithm based on the proposed RSSI distance correction and prediction method. Depending on the correction factor, the correction exponent and the mean distance of the selected distances in each beacon node, the enhanced centroid positioning algorithm is capable of correcting the real-time RSSI based distances and using those corrected distances as an input to the centroid localization algorithm to calculate the final coordinates of the unknown node. Since each beacon node has a universally unique identifier (UUID), the enhanced centroid localization algorithm can identify the stored correction factors and the mean distances of each beacon uniquely.

**Figure 4:**
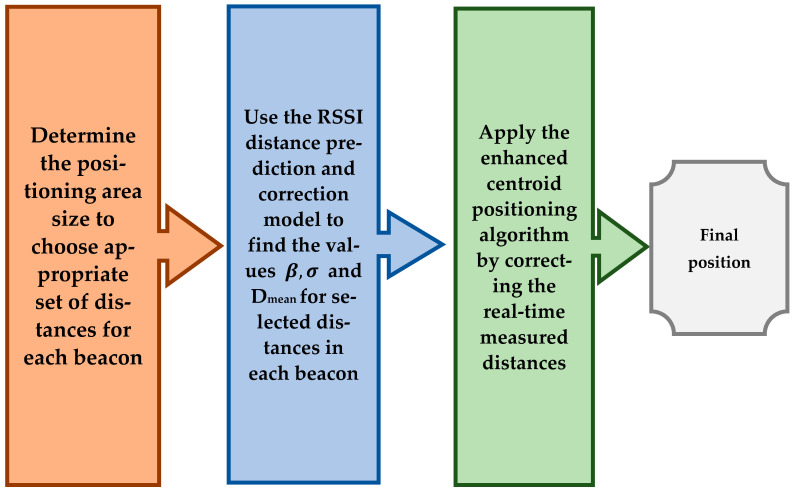
Overall schematic of the proposed positioning technique.

The process of the enhanced centroid localization algorithm is described as follows: the unknown terminal collects RSSI samples from each beacon node. The RSSI samples are converted to equivalent distances using the RSSI log-distance distribution relationship expressed in Equation (3). The mean value of the equivalent distances is obtained from Equation (6) and the smallest three mean distance values are chosen to represent the nearest three beacons to the unknown node. As the source of the three mean distance values is identified by a unique UUID for each beacon, the correction factor and the correction exponent is found by approximating each mean distance value of the three values to the nearest $D_{mean}$ value in the corresponding correction table. The prediction and correction formula used in the unknown node is expressed in Equation (20):

(20) $$D_{corrected}=D_{mean}\cdot \beta^{\sigma }$$

where $D_{mean}$ is the mean value of the equivalent distances from each beacon node; $\beta^{\sigma }$ is the correction factor with its correction exponent based on the approximation of the mean distance value to the nearest $D_{mean}$ value in the corresponding correction table for each beacon UUID; $D_{corrected}$ is the corrected distance between each beacon and the unknown node. Finally, the corrected three distances are treated as an input to the centroid positioning algorithm, where the final unknown node coordinates are obtained. Figure 4 depicts the proposed positioning technique.

**Figure 5:**
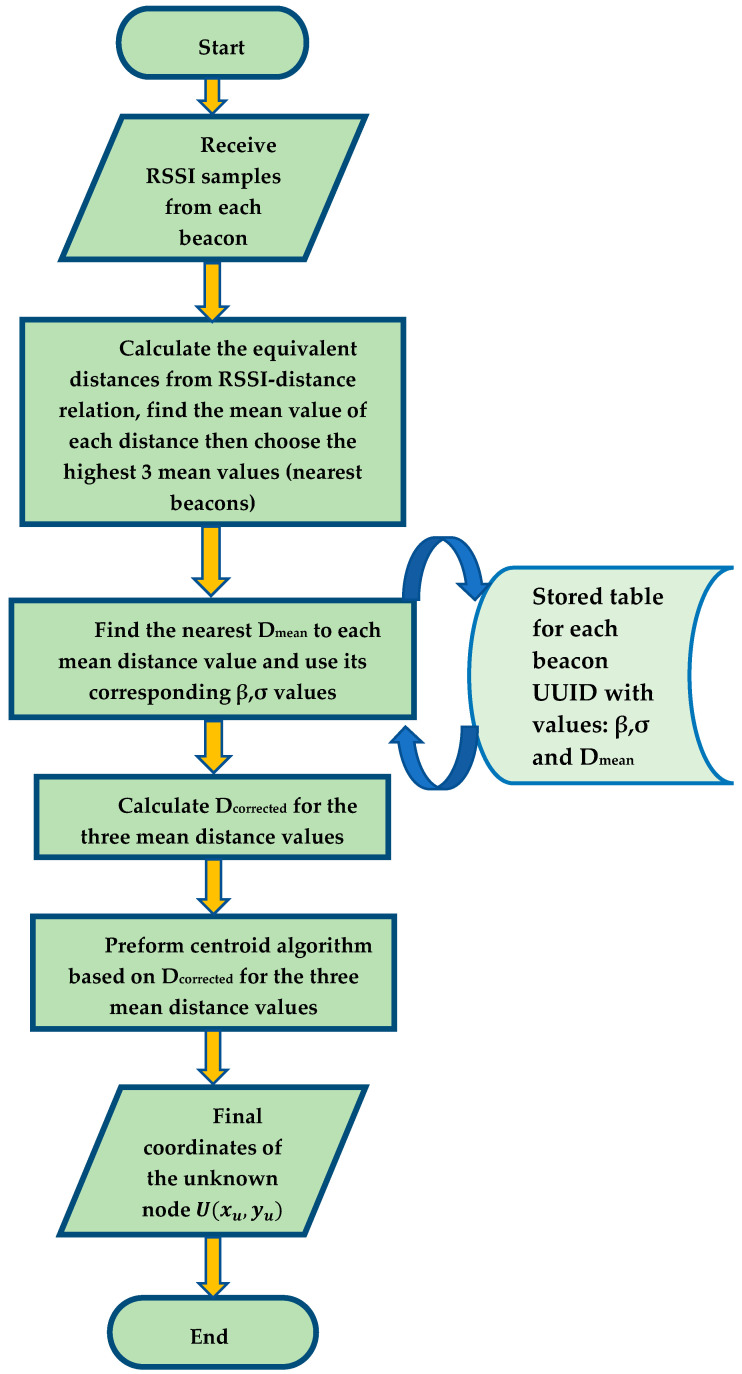
A Flow chart of the proposed enhanced centroid positioning algorithm.

By implementing the previous procedures, the positioning error caused by randomness in RSSI values can be dramatically minimized. Besides, the errors caused by complex indoor environment geometric distribution are minified since the correction factors are driven in the same indoor environment to be positioned. The process of the enhanced centroid positioning algorithm from the perspective of an unknown node is summarized in Figure 5.

## 3. Experiments

In this section, we will display our experiment implementation, including the used software, hardware devices, and the experimental site details.

### 3.1. Device and Software

Our experiment is constructed using five Android terminals, one of them is treated as the unknown node and the other four terminals are acting as BLE iBeacons. Using Beacon Simulator software [41], the Android terminal can be transformed into a virtual BLE beacon transmitter and advertiser. This software offers different beacon configurations to emulate a physical beacon. In this experiment, we used iBeacon platform in all Android terminals to broadcast and advertise iBeacon frames.

**Figure 6:**
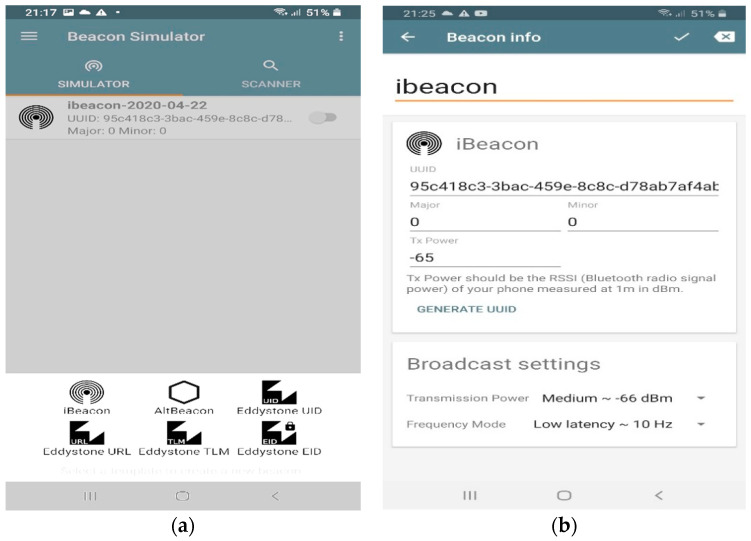
Beacon Simulator application user interface. (**a**) Different beacon platforms; (**b**) iBeacon main configuration used in the experiment [41].

Figure 6 illustrates the application user interface and the iBeacon configuration used in the experiment. The RSSI is continuously measured for one minute at a distance of 1 m from each beacon, and the mean value of the measured RSSI is taken as the value of A in Equation (3). The path loss exponent value was approximated to 3 as a typical value in office indoor environments at 2.4 GHz operating frequency [42]. As can be seen from Figure 6, frequency mode is set to 10 Hz which means that the transmission interval is 0.1 s and hence facilitate our 5000 RSSI samples collection process for the selected distances on each beacon node.

### 3.2. Experimental Sites

**Figure 7:**
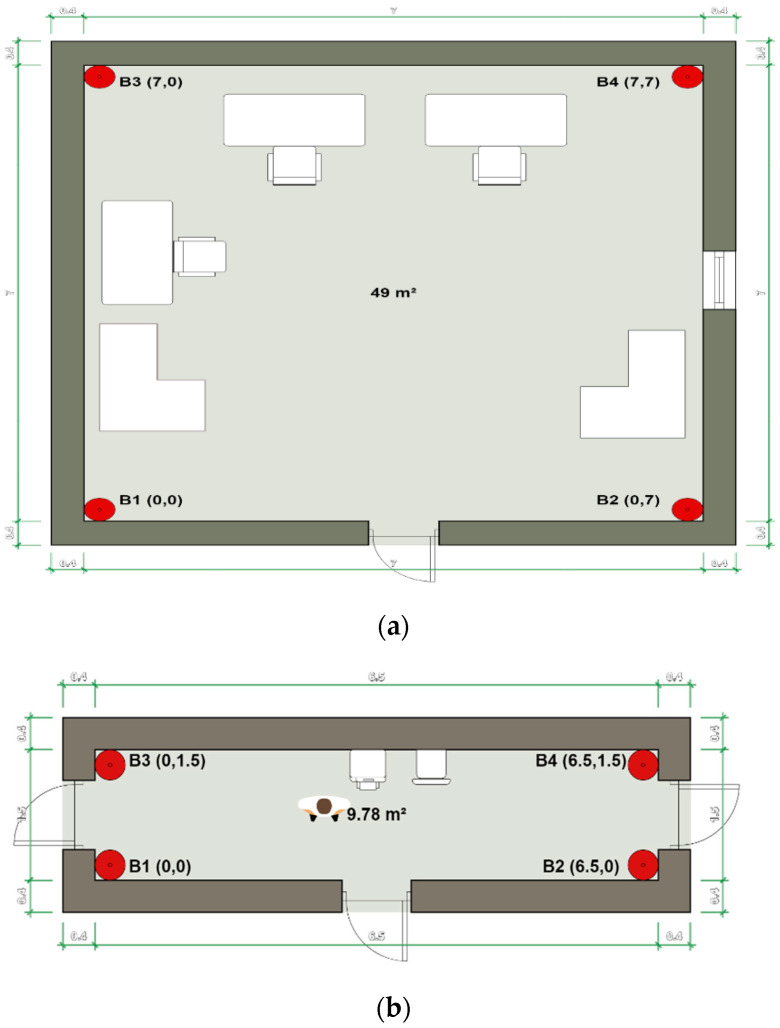
Experimental sites: (**a**) Office room; (**b**) Corridor.

We carried out our experiment in an 7 m ×7 m office room with multiple desks and chairs, and a 6.5 m ×1.5 m corridor with a printer and a single chair. The BLE beacons are placed at the four corners of the office room with coordinates $B_{1}\left(0,0\right),\;B_{2}\left(0,7\right),B_{3}\left(7,0\right)\;\mathrm{and}\;B_{4}\left(7,7\right)$ as illustrated in Figure 7a, and with coordinates $B_{1}\left(0,0\right),\;B_{2}\left(6.5,0\right),B_{3}\left(0,1.5\right)\;\mathrm{and}\;B_{4}\left(6.5,1.5\right)$ for the corridor as depicted in Figure 7b. At the data training stage, 5000 RSSI samples are collected and recorded at 1, 2, 3, 4, 5 and 6 m distances from each beacon node. The RSSI values collection takes place at the experimental sites themselves to consider the multipath and geometric distribution effects on the signal intensity when the correction factors per distance are driven.

The 1, 2, 3, 4 5 and 6 m distances are taken at a 45-degree angle from each corner beacon node. All beacon nodes are placed at the same height at 1.3 m above the ground. Moreover, some people walk randomly during the data training stage and the positioning stage to emulate a real case scenario.

## 4. Results and Discussion

This section presents our experimental results of the proposed positioning technique compared to other positioning algorithms from the localization accuracy and positioning stability point of view. Moreover, the proposed enhanced centroid algorithm performance is evaluated when a different number of RSSI samples are collected in real-time to ensure the proposed technique’s feasibility.

### 4.1. The Correction Factors of the Proposed RSSI Distance Prediction and Correction Model

We used a Bluetooth scanner application, namely Beacon Scanner [43], with no delay between each scan, and the logging frequency is adjusted to be every scan. Then a logging endpoint is recording the received RSSI values for the correction factors’ derivation. We collect the RSSI Values for each distance independently at each beacon, and the values are recorded and sent to a weblogger (logging endpoint). The process of receiving and collecting the RSSI Values takes around 12 min average time at each distance. It worth noting that at 10 Hz mode, two consecutive readings would have precisely the same RSSI values as they could come from the same sensor readings. This means the 5000 samples could come from a much lower quantity of sensor readings (Beacons). This duplication in RSSI values does not affect the proposed technique correction factors’ accuracy as the number of RSSI samples is relatively large. The outcomes of our proposed RSSI distance prediction and correction method at the two experimental sites are shown in Table 2 and Table 3.

As can be seen from Table 2 and Table 3, the mean distance, correction factor, and correction exponent are calculated and driven at 1, 2, 3, 4, 5 and 6 m distances using Equations (6), (7), (9) and (13), respectively. Each beacon is used to calculate its correction factors based on the previously mentioned technique. Those tables are saved on the unknown terminal as it is used later in the enhanced centroid positioning algorithm to correct the real-time RSSI equivalent distances.

### 4.2. Predicted Distances Accuracy

Our proposed RSSI distance prediction and correction model improves the accuracy of the RSSI estimated distances. To verify this, the unknown node is placed at predefined distances from each BLE beacon to test the correction formula and the correction factors. The unknown Android terminal uses the Android Beacon Library [44] to interact with beacons. This library uses RSSI average values to calculate the unknown distances. Therefore, the average RSSI filter is used as a conventional method to calculate the distance between the unknown node and other beacons before applying the positioning algorithm and hence used in our comparison.

**Figure 8:**
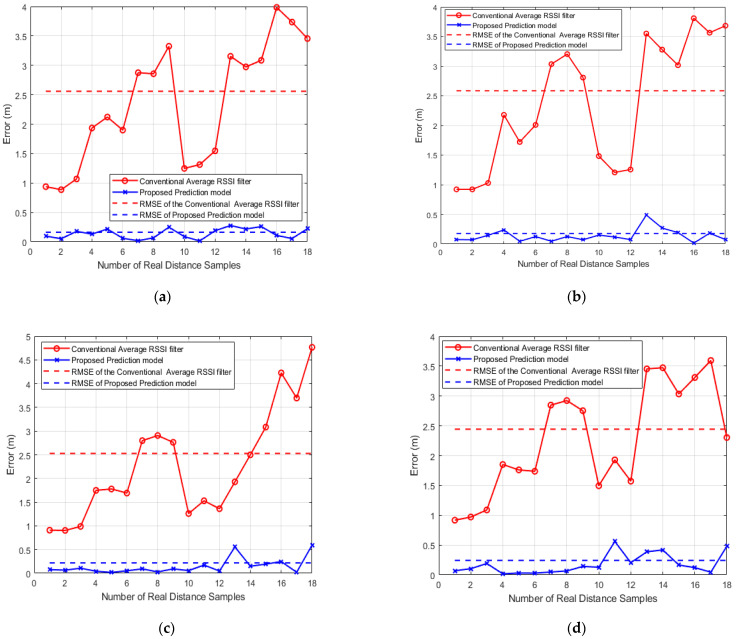
Error and RMSE in the measured distances based on RSSI average filter vs. Proposed RSSI distance prediction model at: (**a**) Beacon 1 (B1); (**b**) Beacon 2 (B2); (**c**) Beacon 3 (B3); (**d**) Beacon 4 (B4).

Figure 8 shows the error in the measured distances using the RSSI averaging method and the predicted distances using the correction factor and the correction exponent based on Equation (20). We took 18 different distances from each beacon to test the proposed correction formula compared with the mean RSSI filter estimated distances. In addition, the root-mean-square error (RMSE) of the measured distances is calculated to compare the proposed prediction method and the conventional average method.

As shown in Figure 8, using the correction factors and correction exponents to correct the measured distance based on our RSSI distance prediction model decreases the error significantly. The RMSE values obtained from the RSSI average filter at the predefined distances are 2.559, 2.585, 2.528 and 2.444 m while the RMSE values obtained from the proposed correction method are 0.163, 0.175, 0.219 and 0.241 m at beacons B1, B2, B3, and B4, respectively. Hence our proposed technique can extremely decrease the errors resulting from RSSI values uncertainty and thus the positioning error can be reduced.

### 4.3. Localization Accuracy

Next, a quantitative analysis of the positioning errors of different positioning algorithms is constructed. We compared our enhanced centroid positioning algorithm based on the proposed RSSI distance prediction and correction model with other positioning algorithms, namely, the triangle centroid algorithm, the weighted triangle centroid algorithm, and the iterative centroid positioning algorithm which were previously discussed.

**Figure 9:**
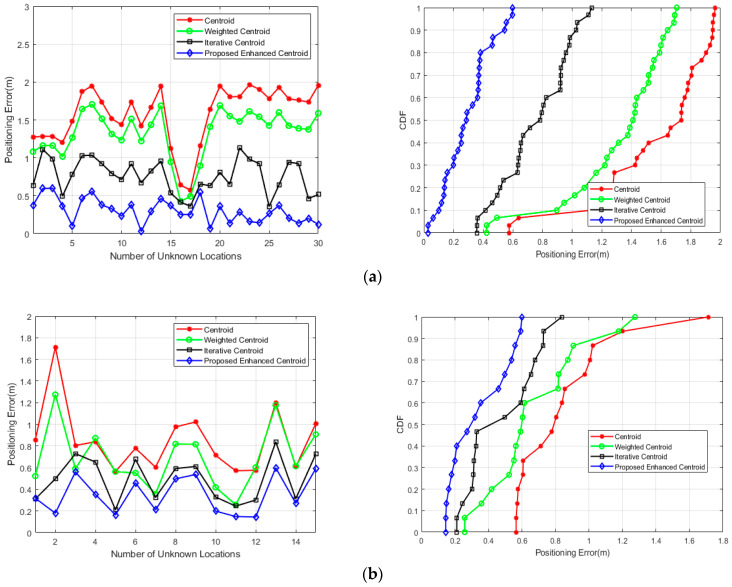
Positioning errors and cumulative distribution function (CDF) of the positioning error of the proposed enhanced centroid algorithm and the other positioning algorithms: (**a**) The office room; (**b**) The corridor.

In the positioning stage, the unknown node (Android terminal) was placed in 30 different locations inside the office room and 15 different locations in the corridor to calculate its final positioning coordinates. Figure 9 shows the positioning errors in meters and the cumulative distribution function (CDF) of the positioning error as recommended by the ISO18305:2016 standard for real-time locating systems [45] for the 30 locations (office room) and the 15 locations (corridor) using different localization algorithms, including our enhanced centroid algorithm.

It is evident from the figure that almost all the errors obtained by using the enhanced centroid positioning algorithm based on the proposed RSSI distance correction model were less than the other positioning algorithms. It is important to mention that the other positioning algorithms use the measured distances between the unknown terminal and other beacon nodes based on averaging the received RSSI samples at the Android terminal.

**Figure 10:**
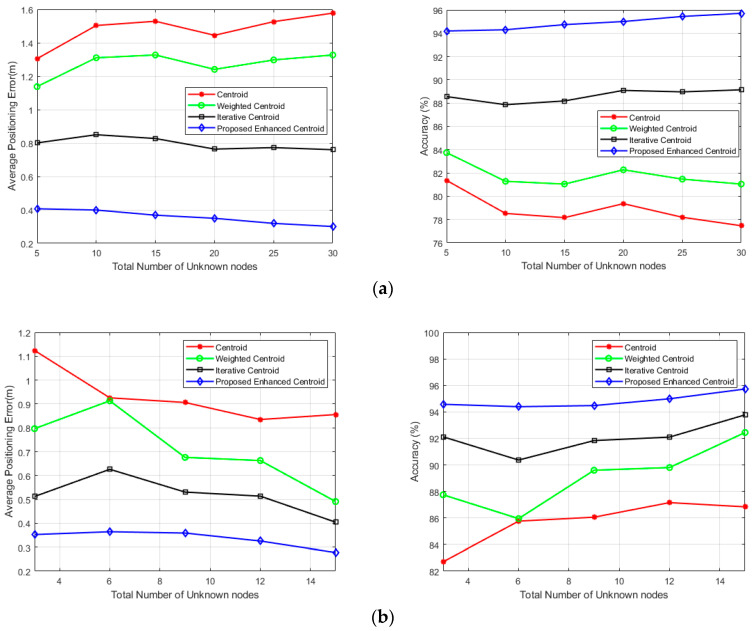
The number of unknown locations and its corresponding Average positioning error and Accuracy percentage: (**a**) The office room; (**b**) The corridor.

In Figure 10, the average localization error (ALE) and the percentage of accuracy are shown with respect to total unknown locations ranging from 5 to 30 unknown location (office) and from 3 to 15 unknown location (corridor).

As shown on the left in Figure 10a,b, the average localization error values of the proposed enhanced centroid algorithm at both the office room and the corridor are considerably less than the centroid, weighted centroid, and iterative centroid algorithms. The average localization error is obtained at the office room for 5, 10, 15, 20, 25, and 30 unknown nodes and the corridor for 3, 6, 9, 12, and 15 unknown nodes. As also can be seen in Figure 10a,b on the right, the accuracy percentage of the proposed enhanced centroid algorithm at the office room ranges from 94.19% to 95.707%, while its range changes from 77.465% to 81.358%, 81.042% to 83.74%, and 87.859% to 89.14% for the centroid, weighted centroid and iterative centroid algorithms, respectively. The accuracy percentage of the proposed enhanced centroid algorithm at the corridor ranges from 94.57% to 95.776%, while its range changes from 82.7% to 87.15%, 85.95% to 92.44%, and 90.367% to 93.777% for the centroid, weighted centroid and iterative centroid algorithms, respectively.

Table 4 summarizes the localization accuracy of the four positioning algorithms at the two experimental sites. Hence the enhanced centroid algorithm based on the proposed RSSI distance prediction and correction model outperforms the other positioning algorithms in terms of localization accuracy.

**Figure 11:**
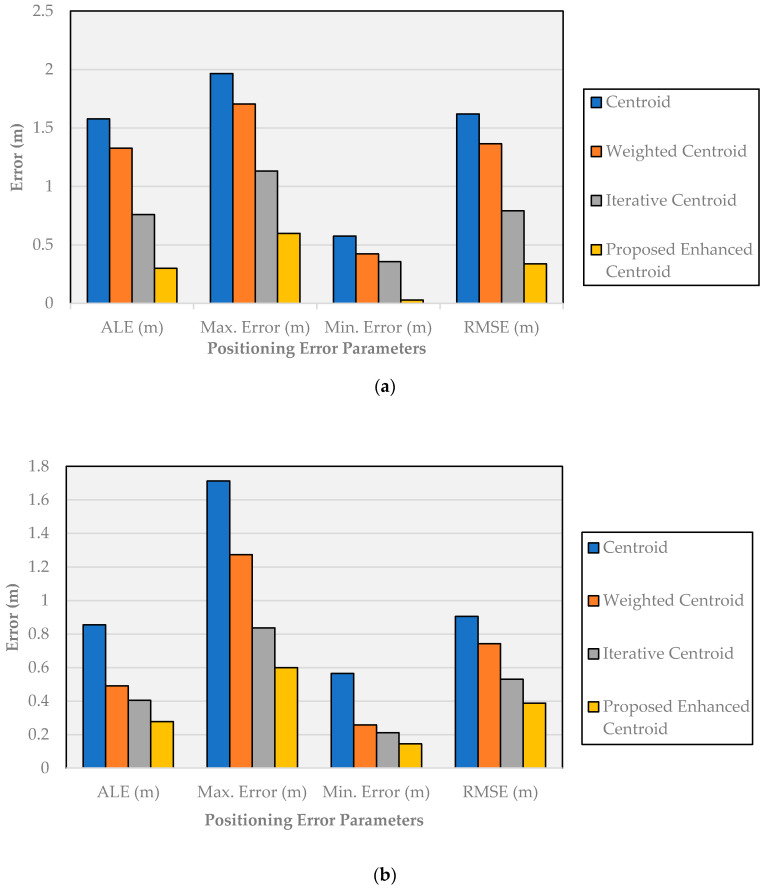
Positioning error parameters of different positioning algorithms: (**a**) The office room; (**b**) The corridor.

The positioning error parameters of the proposed enhanced centroid algorithm and the other positioning algorithms, including the average localization error (ALE), maximum error, minimum error, and the RSME at both experimental sites, are presented in Figure 11.

**Figure 12:**
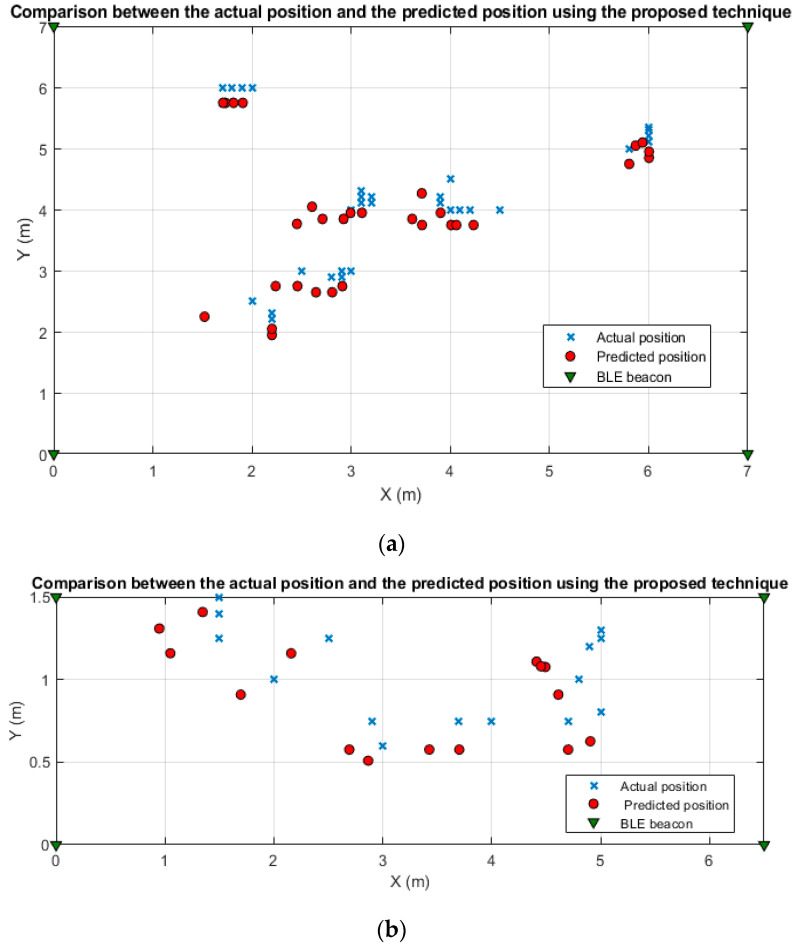
Comparison between the actual test points position and the predicted position using the proposed enhanced centroid algorithm at: (**a**) The office room; (**b**) The corridor.

As depicted in Figure 11a, the RMSE, maximum error, and minimum error values of the positioning results obtained by the enhanced centroid positioning algorithm based on the proposed RSSI distance prediction and correction model at the office room are lower than the other positioning algorithms. Moreover, the average localization errors of the four algorithms are 0.3, 0.76, 1.327 and 1.577 m, respectively, and the localization accuracy of the proposed enhanced centroid algorithm is improved by 80.97%, 77.39% and 60.526% compared with the centroid algorithm, the weighted centroid algorithm and the iterative centroid algorithm, respectively. As depicted in Figure 11b, the four algorithms’ average localization errors at the corridor are 0.278, 0.404, 0.491 and 0.855 m, respectively. The proposed enhanced centroid algorithm’s localization accuracy is improved by 67.51%, 43.42%, and 31.3% compared with the centroid algorithm, the weighted centroid algorithm, and the iterative centroid algorithm, respectively. Finally, Figure 12 presents a comparison between the actual test points position and the predicted position using the proposed enhanced centroid algorithm at the two experimental sites.

Thus, it can be clarified that the enhanced centroid localization algorithm based on the proposed RSSI distance prediction and correction method dramatically improves the positioning accuracy in indoor environments. Our proposed method can be used in small and medium indoor environments, especially at room level. However, the limitation of the proposed method enlarges in wide indoor environments since more beacon nodes are required to cover the localized area; accordingly, more pre-trained distances are needed to obtain correction factors at the expense of time factor.

### 4.4. Positioning Stability

**Figure 13:**
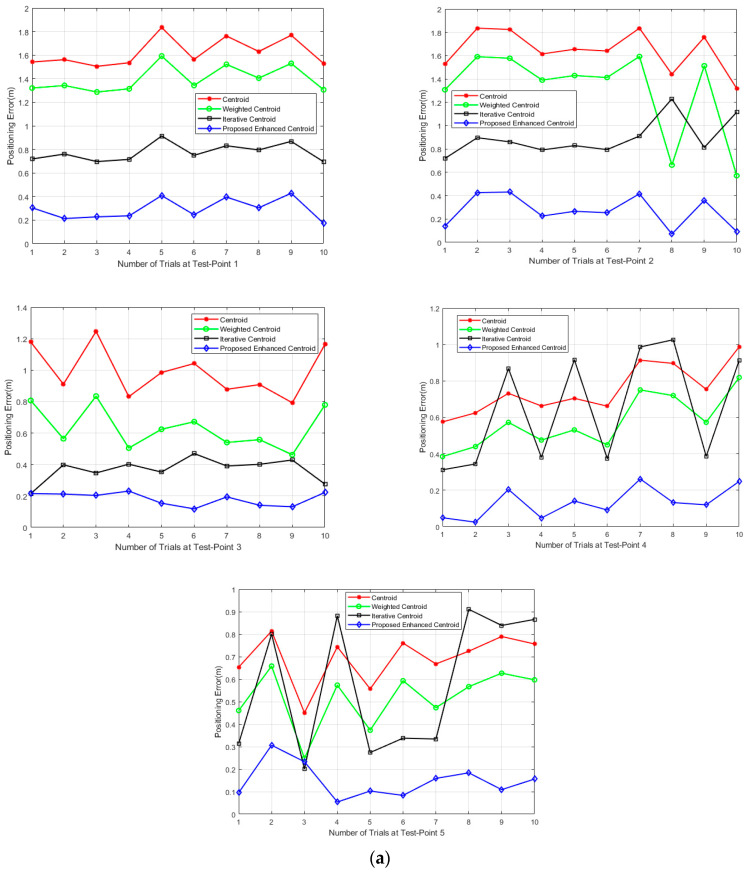

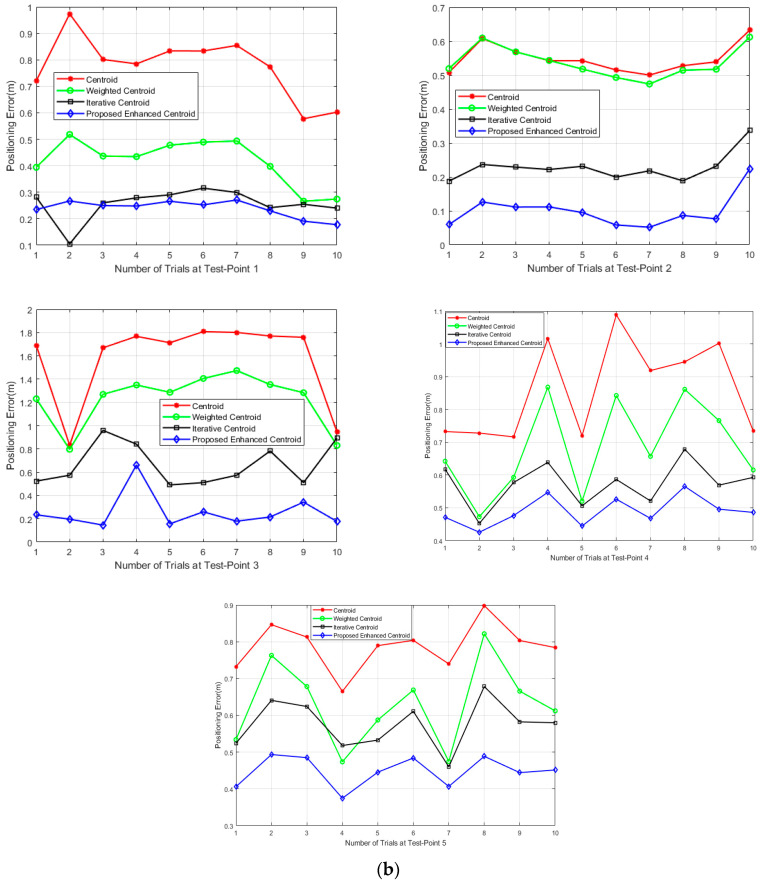
Positioning error comparison of the proposed positioning technique and the other positioning algorithms deploying the average RSSI filter at 5 test points for 10 trials: (**a**) The office room; (**b**) The corridor.

Figure 13 presents the calculated positioning errors in sequence. The positioning error is calculated 10 times at random five test points in both the office and the corridor. Based on each test point’s accuracy fluctuations, the proposed positioning technique’s errors vary little, while the errors by the other positioning algorithms using average RSSI filter change significantly. Fortunately, the proposed technique also almost gains the best accuracy in every positioning trial’s overall improvement.

The overall positioning stability is measured using the sample standard deviation (SSD); the SSD can be obtained using the following formula:

(21) $$SSD=\;\frac{STD\left(E\right)}{MEAN\left(E\right)}$$

where STD(_) is the standard deviation, MEAN(_) is the average value; E is the positioning error for 10 times at each test-point.

Table 5 and Table 6 show that the proposed positioning technique achieves smaller sample variance and closer average positions for each test point than the other positioning algorithms deploying average filter.

The office room’s overall positioning stability is 1.64, 2.077, and 2.14-fold better for the proposed enhanced centroid than the centroid, weighted centroid, and iterative centroid, respectively. The corridor’s overall positioning stability is 2.3, 1.94, and 1.29-fold better for the proposed enhanced centroid than the centroid, weighted centroid, and iterative centroid, respectively. Thus, we can claim that our enhanced centroid algorithm based on the proposed RSSI distance correction model improves indoor environments’ overall positioning stability. The reason for that is due to the low deviation characteristics of proposed RSSI distance correction measurements as early discussed in Section 2.2 in Table 1.

### 4.5. The Effect of Different RSSI Samples’ Number on the Localization Accuracy

In this experiment, the proposed enhanced centroid algorithm was performed at 5 random test points in both experimental sites. The proposed enhanced centroid algorithm was performed at each point when a different number of RSSI samples is collected. The proposed algorithm was conducted 100 times for each number of collected RSSI samples, and the average error of the 100 trials was computed. Table 7 and Table 8 presents the proposed technique’s localization error at the office room and the corridor when a different number of RSSI samples is collected.

The proposed enhanced centroid algorithms’ overall average localization error at the five test points varies from 0.313 to 0.337 m and from 0.326 to 0.354 m at the office room and corridor, respectively, when the number of received RSSI samples varies from 100 to 50 RSSI sample. It can be inferred that the received RSSI samples’ number has no great impact on the localization accuracy of the proposed technique. However, if the RSSI samples’ number becomes lower than the 50 RSSI sample, 40 RSSI samples, such as or below, the proposed enhanced centroid algorithm sometimes becomes infeasible. That is it, the RSSI samples’ number is not enough to correctly calculate the proposed model correction factors, and the corrected estimated distance becomes smaller than the real distance. Therefore, it is recommended to collect at least 50 RSSI samples or more at the unknown node to ensure the feasibility of the proposed correction model.

## 5. Conclusions

Due to the rapid development of wireless communication technology, the position information of nodes has become a critical feature in different applications. Indoor localization techniques face common issues, including poor localization accuracy, expensive communication costs and high energy consumption. A new cost-effective and high accuracy and stability localization solution is presented in this paper to overcome these problems. The proposed RSSI distance prediction and correction model introduced new correction factors to accurately predict the real distance between the unknown terminal and the anchor (beacon) nodes. Importantly, our practical results provide evidence for the correctness of the estimated distances based on the proposed RSSI distance correction model.

Moreover, the experimental results of the enhanced centroid localization algorithm based on the proposed RSSI distance prediction and correction model shows a significant improvement in the positioning accuracy of the unknown nodes. The proposed enhanced centroid algorithm’s localization accuracy is improved by 80.97% and 67.51% in an office room and a corridor, respectively, compared with the traditional RSSI positioning algorithm. The proposed technique also promotes positioning stability by 1.64 and 2.3-fold at the office room and the corridor, respectively, compared to the conventional RSSI positioning method. The proposed correction model is entirely feasible in real-time when the RSSI sample number is 50 or more. However, if the received RSSI samples are less than 50, the proposed technique is partially feasible. Finally, the proposed technique is intended for small to medium indoor environments, especially at the room level. In the future, we can utilize the proposed method in trajectory planning and navigation of objects moving at high speed in larger indoor environments.

## Figures and Tables

**Table 1:** The standard deviation of the collected RSSI samples and their equivalent distances.

Real Distance (m)	Standard Deviation	
	RSSI Samples (dBm)	Distance Samples (m)
1	3.32	0.547
2	2.642	0.792
3	2.658	1.258
4	2.571	1.171
5	2.576	1.802
6	3.017	2.281

**Table 2:** The output factors of the RSSI distance prediction and correction model from one beacon at the office room.

Real Distance (m)	Correction Exponent (*σ*)	Correction Factor (*β*)	Mean Distance in Meters (*D_mean_*)
1	7.114	0.917	2.014
2	21.993	0.97	3.863
3	19.561	0.964	6.085
4	15.285	0.993	5.519
5	11.109	0.959	8.281
6	130.354	0.996	10.039

**Table 3:** The output factors of the RSSI distance prediction and correction model from one beacon at the corridor.

Real Distance (m)	Correction Exponent (*σ*)	Correction Factor (*β*)	Mean Distance in Meters (*D_mean_*)
1	6.568	0.91	1.847
2	26.654	0.975	3.883
3	28.65	0.979	5.411
4	11.913	0.993	0.965
5	12.282	0.963	7.943
6	78.746	0.993	9.999

**Table 4:** The localization accuracy percentage range of the four positioning algorithms at the two experimental sites.

Experimental Site	Localization Accuracy Percentage Range			
	Centroid	Weighted Centroid	Iterative Centroid	Proposed Enhanced Centroid
Office Room	77.46–81.36%	81.042–83.74%	87.86–89.14%	94.19–95.707%
Corridor	82.7–87.15%	85.95–92.44%	90.36–93.78%	94.57–95.776%

**Table 5:** The sample standard deviation (SSD) of the four positioning algorithms at five test points in the office room.

Test Point Number	Sample Standard Deviation (SSD) in Meters			
	Centroid	Weighted Centroid	Iterative Centroid	Proposed Enhanced Centroid
1	0.12	0.088	0.058	0.031
2	0.176	0.046	0.042	0.05
3	0.157	0.229	0.181	0.153
4	0.137	0.143	0.066	0.044
5	0.113	0.115	0.066	0.041
**Average SSD**	0.141	0.178	0.184	0.086

**Table 6:** The sample standard deviation (SSD) of the four positioning algorithms at five test points in the corridor.

Test Point Number	Sample Standard Deviation (SSD) in Meters			
	Centroid	Weighted Centroid	Iterative Centroid	Proposed Enhanced Centroid
1	0.117	0.088	0.058	0.032
2	0.043	0.046	0.042	0.05
3	0.365	0.229	0.181	0.153
4	0.147	0.143	0.066	0.044
5	0.064	0.115	0.066	0.041
**Average SSD**	0.147	0.124	0.083	0.064

**Table 7:** The localization errors of the proposed technique at five test points for different RSSI samples’ number in the office room.

RSSI Samples’ Number		Localization Error (m)				
	Test-Point 1	Test-Point 2	Test-Point 3	Test-Point 4	Test-Point 5	Overall ALE
100 RSSI	0.311	0.286	0.223	0.432	0.312	0.313
90 RSSI	0.331	0.29	0.203	0.425	0.322	0.314
80 RSSI	0.305	0.303	0.217	0.434	0.326	0.317
70 RSSI	0.312	0.307	0.22	0.438	0.316	0.319
60 RSSI	0.329	0.32	0.235	0.422	0.33	0.327
50 RSSI	0.352	0.32	0.233	0.442	0.341	0.337

**Table 8:** The localization errors of the proposed technique at five test points for different RSSI samples’ number in the corridor.

RSSI Samples’ Number		Localization Error (m)				
	Test-Point 1	Test-Point 2	Test-Point 3	Test-Point 4	Test-Point 5	Overall ALE
100 RSSI	0.411	0.445	0.144	0.354	0.278	0.326
90 RSSI	0.411	0.455	0.156	0.361	0.286	0.334
80 RSSI	0.417	0.455	0.159	0.368	0.291	0.338
70 RSSI	0.418	0.467	0.165	0.381	0.288	0.344
60 RSSI	0.415	0.465	0.176	0.382	0.293	0.346
50 RSSI	0.423	0.468	0.193	0.386	0.3	0.354

## Data Availability

Data sharing not applicable.
